# Understanding the interplay between video game design features and dysregulated gaming patterns: A call to anchor future research directions in interactionist frameworks^☆^

**DOI:** 10.1016/j.abrep.2025.100609

**Published:** 2025-04-17

**Authors:** Maèva Flayelle, Mélina Andronicos, Daniel L. King, Joël Billieux

**Affiliations:** a Institute of Psychology, University of Lausanne, Lausanne, Switzerland; b Center for Excessive Gambling, Addiction Medicine, Lausanne University Hospital (CHUV), Lausanne, Switzerland; c College of Education, Psychology & Social Work, Flinders University, Adelaide, Australia

## Abstract

• Research on video game design and dysregulated gaming needs further development.
• Research directions must shift from linear causal approaches to interactionist ones.
• Relevant research directions, with examples of suitable study designs, are proposed.

## 1 Introduction

As a massively popular form of digital entertainment all around the world, video gaming attracts billions of individuals through an extensive range of games across a variety of platforms and devices (Statista, 2024). For most people, playing video games is associated with a range of benefits — from relaxation and enjoyment to fulfilment of needs related to social belonging or autonomy (Granic et al., 2014, Halbrook et al., 2019). However, research shows that a minority of gamers experience difficulties in regulating their engagement in gaming activities, which can result in mental and physical symptoms as well as functional impairment (e.g., Reed et al., 2022, Rumpf et al., 2018). Meta-analytic studies suggest that the prevalence of gaming disorder is around 1.5 to 2 % of the general population (Kim et al., 2022, Stevens et al., 2021), while other empirical studies have reported an increase in treatment-seeking gamers (e.g., Han et al., 2018, Rumpf et al., 2018). Based on this scientific evidence, the World Health Organization officially recognized gaming disorder as a “disorder due to addictive behaviour” in the 11th revision of the International Statistical Classification of Diseases and Related Health Problems in 2019 (ICD-11; World Health Organization, 2019). According to the ICD-11, gaming disorder is characterized by impaired control, increasing priority given to gaming over other activities, and continued gaming despite negative consequences in important areas of functioning. Research emphasizes the centrality of impaired control as a mechanism contributing to negative outcomes in gaming. For example, a recent study found that impaired control is positively related to gaming-related harm across financial, psychological, social, relational, and functional domains in individuals with self-identified gaming disorder, even when accounting for other psychological and demographic factors (Kowalik et al., 2024).

The formal recognition of gaming disorder as a mental condition has prompted further exploration of players’ psychological characteristics contributing to the emergence and persistence of problem gaming symptoms, including sociodemographic attributes, genetic risk factors, motivational tendencies, personality traits, neurobiological processes, and co-occurring mental health issues (Király et al., 2023). Compared with the significant efforts invested in many of these research areas, however, empirical investigations into how certain design features of video games could foster diminished control over gaming behaviors has been sparse (King et al., 2019a, Rehbein et al., 2024, Saini and Hodgins, 2023). Yet, studies’ results not only agree that, in comparison with offline games, online types of games are linked to higher prevalence rates of uncontrolled and problematic gaming (Lemmens and Hendriks, 2016, Männikkö et al., 2017), but also that certain game genres — notably Massively Multiplayer Online Role-Playing Games (MMORPGs), first-person shooter games, and real-time strategy/Multiplayer Online Battle Arena (MOBA) games — are more strongly associated with elevated players’ engagement and dysregulated gaming patterns than other game types (King et al., 2019a, Rehbein et al., 2021). Crucially, however, it is important to emphasize that while these genres are generally linked to problematic gaming patterns, this is likely due to specific game design features (e.g., immersive environments, reward systems, social interaction mechanics) rather than the game genre itself (King et al., 2010, King et al., 2019b, Klemm and Pieters, 2017, Rehbein et al., 2021). This suggests the need for a stronger research emphasis on understanding the interplay between design features of video games (beyond overarching game types or genres) and diminished control over gaming. It should be acknowledged, however, that several theoretical taxonomies have been published in areas of psychological research, which may serve as conceptual foundations pertinent to this objective.

### 1.1 Design features of games relevant to elevated players’ engagement and dysregulated gaming patterns: Existing taxonomical models

One of the earliest gaming taxonomies was proposed by Wood and colleagues (2004) who developed a framework of structural characteristics of video games that could promote player’s enjoyment and sustained engagement. This framework consists of a list of thirteen distinct groupings of features including: a) *sound*, b) *graphics*, c) *background and setting*, d) *duration of game*, e) *rate of play*, f) *advancement rate*, g) *use of humour*, h) *control options*, i) *game dynamics*, j) *winning and losing features*, k) *character development*, l) *brand assurance*, and m) *multiplayer features*.

King and colleagues (2010) then expanded upon this framework and proposed a five-feature model comprising a total of 24 video game structural characteristics that may contribute to excessive gaming, which were divided into the five following categories: a) *social features*, b) *manipulation and control features*, c) *narrative and identity features*, d) *reward and punishment features*, and e) *presentation features*. More than a decade later, additional taxonomies were introduced. Drawing from a scoping review of the literature on the associations between design features of video games and problematic gaming, Saini and Hodgins (2023) outlined a revised version of King and colleagues’ taxonomical model involving only two broader classes of characteristics: a) *structural features enhancing in-game immersion and realism,* and b) *gambling-like structural features.*

Based on a literature review of the relationships between technology design features and loss of control experienced by individuals in the context of various online activities, Flayelle and colleagues (2023) developed a general taxonomy of design features of online applications that may promote dysregulated involvement in online behaviors, including video gaming. Relevant to this particular activity, nine video game design features were suggested as especially influential across four categories differentiating between model-free and model-based mechanisms underlying learning and behavioral control: a) *reinforcement schedules promoting habit formation and incentive sensitization* (model-free design features), b) *features contributing to overvaluation of the positive outcomes of the online activity* (model-based design features), c) *partial goal fulfilment* (model-based design features), and d) *features strengthening cognitive biases and distorted beliefs* (model-based design features).

With these early conceptual foundations, there is agreement amongst researchers that empirical research is needed to elucidate which video game design features, or combinations of features, are most likely to foster the development and perpetuation of dysregulated gaming patterns (Flayelle et al., 2023, King et al., 2019a, Rehbein et al., 2021, Rehbein et al., 2024, Saini et al., 2024). In connection with this, several recommendations have been made in terms of research directions to prioritize on this topic, including two commonly proposed avenues, as outlined below.

## 2 Addressing the potential role of video game design features in fostering dysregulated gaming patterns: Two commonly proposed research directions

The first suggested direction for research primarily revolves around employing more sophisticated methodologies and more adequate samples as most of the existing research is limited by its reliance on cross-sectional data collected through retrospective self-report surveys among small, self-selected convenience samples, without any guarantee that these involve participants experiencing impaired control and/or adverse consequences related to their gaming behavior (Griffiths and Nuyens, 2017, King et al., 2019a, Rehbein et al., 2021). It was therefore suggested to adopt other sound approaches such as conducting ecologically valid field experiments, implementing longitudinal study designs, and collaborating with gaming operators to access real-time behavioral tracking data of players, especially adolescents experiencing the early signs of uncontrolled and problematic gaming-related issues and adults with established dysregulated gaming patterns (Griffiths and Nuyens, 2017, Hing et al., 2023, King et al., 2019a, Rehbein et al., 2021, Rehbein et al., 2024).

Then, capitalizing on data obtained through these means, a second key direction for future inquiry commonly outlined by researchers consists of elucidating causal relationships between video game design features and uncontrolled patterns of gaming behavior. This could take the form of 1) gathering an inventory of the design features inherent in the games played by the study participants using, for example, recent assessment tools such as the *Risk Characteristics Checklist for Games* (RCCG; Rehbein et al., 2024) or the *Saini-Hodgins Addiction Risk Potential of Games Scale* (SHARP-G; Saini et al., 2024) — both of which were developed to rate the addictive potential of video games based on their intrinsic structural features —; and 2) examining the differential predictive value of design features for symptoms of problematic gaming via multivariate regression analyses or supervised machine learning approaches (Hing et al., 2023, Rehbein et al., 2024, Saini et al., 2024).

It must be stressed, however, that this proposed research path, merely aimed at isolating the causal relevance of specific video game design features to the onset and progression of uncontrolled and problematic patterns of gaming behavior, remains insufficient to capture the essence of the relationship. Indeed, by constraining the research focus on the influence of these characteristics on gaming behaviors, such a direction also implicitly (and inadvertently) conveys the simplistic view that these would have direct and uniform effects on gamers. At the current early stage of empirical research on the issue, it is thus crucial to recall that investigation of the interplay between video game design features and diminished control over gaming needs to go beyond mere linear causal approaches (i.e., linking specific features to particular gaming outcomes or attributing dysregulated gaming to certain “addictive” characteristics of games), which, as a matter of fact, are considered completely outdated from the standpoint of research on the psychology of media use and its effects.

### 2.1 The insufficiency of analyzing media effects through simplistic causal chains

Far from being uniform or deterministic, media effects are instead complex and contingent on a wide variety of personal, social, and cultural factors. Contemporary media effects theories, such as the Uses and Gratifications Theory (Katz et al., 1973, Rubin, 2009), do not consider users as passive recipients for whom properties of the consumed media straightforwardly exert effects or influence. Rather, they emphasize users’ active role in deliberately selecting specific media to satisfy personal needs, thus implying significant variations in media effects on individuals according to the unique interactional dynamics occurring between media content and user characteristics. Similarly, the Differential Susceptibility to Media Effects Model (Valkenburg & Peter, 2013) highlights how media effects rely on individual susceptibility influences, including dispositional, developmental, and social factors. Therefore, just as there are strong individual differences in susceptibility to media effects, it can be argued that video game design features are very unlikely to affect every gamer in the same manner or to universally promote diminished control over gaming.

It is also noteworthy that some recent etiological models describing the development and maintenance of gaming disorder point in the same direction. For example, the I-PACE (Interaction of Person-Affect-Cognition-Execution) model (Brand et al., 2019) conceptualizes addictive behaviors involving the use of any online applications (e.g., online gaming and gambling, cybersex, social networking) as resulting from a dynamic interplay between individual predisposing factors (e.g., person’s core characteristics, driving motives), affective and cognitive responding styles (e.g., coping and mood regulation strategies, attentional biases), and reduced executive functioning and inhibitory control. Central to the addictive process according to this interactive framework is the varying role of gratification and compensation experiences (through positive and negative reinforcement) while performing the behavior. As the model evolves and becomes increasingly specified (Brand et al., 2025), clarifying how some individuals may be particularly prone to experience positive/negative reinforcement through specific (video game) design features should also be considered. In a similar vein, other theoretical models such as the Pathway Model of Problematic Mobile Phone Use (Billieux et al., 2015a, Billieux et al., 2015b) or the Pathways Model of Problem Gambling (Nower et al., 2022) emphasize that different pathways can lead to addictive behaviors, each of which is influenced by specific individual psychological characteristics as risk factors (e.g., poor inhibitory control for the impulsive pathway). While these models are resolutely person-centered, they may also greatly benefit from integrating design feature perspectives to clarify not only who is at risk, but also how and when specific features interact with psychological vulnerabilities to reinforce maladaptive patterns of use.

**Fig. 1:**
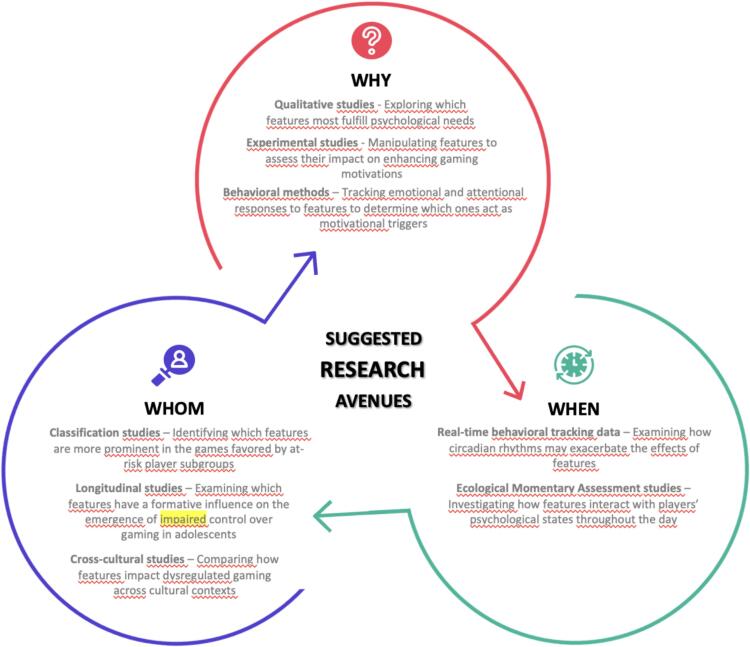
Suggested research avenues for investigating the interplay between video game design features and dysregulated gaming patterns.

Therefore, while many researchers rightly state that investigating the role of video game design features in dysregulated gaming will improve understanding of the interaction between game features and individual vulnerability factors (King et al., 2019a, Larrieu et al., 2023, Rehbein et al., 2021, Rehbein et al., 2024, Saini et al., 2024), we contend that such an interactionist approach must be fully reflected in related proposed research avenues. That is necessary to account for the complexity of individual differences, motivations, psychological states, and contextual influences that mediate and moderate the effects of video game design features in promoting diminished control over gaming. To put it differently, better understanding this interplay must inevitably entail systematizing the potential effects of these features in upcoming research initiatives, the projected focus of which needs to shift from “which video game design features facilitate dysregulated gaming patterns” to a more sophisticated and multi-dimensional perspective addressing “which, why, when, and for whom video game design features facilitate dysregulated gaming patterns”. Accordingly, the following section outlines related directions for future research on this topic, including examples of suitable study designs, all of which is summarized in Fig. 1.

## 3 Suggested research avenues for examining the influence of video game design features in a multicausal and systematic way

### 3.1 Understanding “why” certain video game design features may contribute to diminished control over gaming

As users’ active agency in selecting and engaging with media content is paramount to understanding how media effects unfold, this recommended research focus primarily involves investigating how video game design features align with or enhance players’ motivations for gaming, some of which being known to be more strongly associated with problematic gaming (e.g., escapism, social interaction or achievement; Bäcklund et al., 2022, King and Delfabbro, 2009, Melodia et al., 2022). To this end, conducting qualitative studies (such as focus groups and in-depth interviews) among problematic gamers is needed to explore which types of video game design features (e.g., reward systems or social connectivity features) are perceived as contributing the most to fulfill personal psychological needs (e.g., competence or social connections), and to investigate their links with dysregulated gaming. It should be stressed, however, that there is no one-to-one correspondence between psychological needs and dysregulated gaming behaviors. Rather than acting as direct causes, these motivational drivers are indeed only likely to interact with a range of individual, contextual, and game-related factors to influence gaming engagement. Experimental studies in which specific game features (e.g., sensory features) would be altered or manipulated through different versions of a game must be also conducted to observe their impact on gaming engagement (e.g., comparing players’ levels of immersion using self-reports and physiological measures). Another promising direction is to employ behavioral methods such as emotional response tracking (e.g., using a facial recognition software) and eye tracking, to record both emotional and attentional reactions of players while interacting with games involving highly engaging mechanics, such as progression features (e.g., leveling up) or competitive ranking systems (e.g., leaderboards). This approach will allow us to determine which of these elements actually trigger stronger emotional highs and lows, which may reinforce achievement-oriented motivation and overall engagement/retention.

### 3.2 Examining “when” specific video game design features are the most influential to the development of dysregulated gaming

This research pathway involves examining contextual and situational factors that may exacerbate the effects of video game design features in facilitating dysregulated gaming patterns. Likely to be not merely static, the extent of their impact might depend on the interrelation of contextual influences, psychological states, and circadian rhythms, all of which vary throughout the day. For example, it may be that time-limited features in games — leading players to act impulsively or on the spur of the moment — go hand in hand with an ever-increasing susceptibility to impaired self-regulation as the day progresses and available cognitive resources diminish. Along a similar vein, turning to gaming to unwind after a stressful day may make immersive characteristics (e.g., narrative, music, time distortion features) particularly engaging. Therefore, the temporal dynamics underlying these effects should be investigated through studies accessing real-time data from gaming platforms to examine how gaming during different times of the day precisely connects with video game design features to promote diminished control over gaming. To this end, relying on objective measures of gaming behaviors is important as it has been demonstrated that self-reported assessments poorly reflect actual gaming involvement (Johannes et al., 2021, Larrieu et al., 2023, Parry et al., 2021). As an additional valuable “in-the-moment” approach, Ecological Momentary Assessment (EMA) studies should be conducted among problematic gamers to track their psychological states and gaming behaviors via daily surveys, and then examine how playing games, with and without certain features, interacts with negative emotional states (e.g., stress, depression, anxiety), which are well-established risk factors for uncontrolled and problematic gaming (Gao et al., 2022, King et al., 2019a, Maroney et al., 2019).

### 3.3 Identifying “for whom” particular video game design features have a stronger impact

A number of individual differences have been implicated in the development and maintenance of uncontrolled and problematic gaming, including personal and psychological characteristics (e.g., marked impulsivity, negative affectivity, and loneliness, low levels of self-control, conscientiousness, and self-esteem; Chew, 2022, Gao et al., 2022, Gervasi et al., 2017, Paulus et al., 2018, Ropovik et al., 2023, Şalvarlı and Griffiths, 2019 Warburton et al., 2022), developmental stages (i.e., higher prevalence among adolescents; Lau et al., 2018, Mihara and Higuchi, 2017, Stevens et al., 2021) and cultural influences (i.e., higher prevalence in Asian populations; Kim et al., 2022, Saunders et al., 2017, Stevens et al., 2021). Accounting for individual-level influences relevant to impaired control over gaming, this suggested line of inquiry must thus explore which (and how) video game design features resonate more with different subcategories (or profiles) of vulnerable people. In this regard, several types of research strategies are to be considered. For example, studies capitalizing on a person-centered approach (through e.g., cluster or latent class analyses) aimed at grouping players based on demographic and psychological characteristics, and then assessing the inherent features of the games they play as external correlates, have the potential to distinguish which games characteristics stand out preferentially within subgroups with increased vulnerability to dysregulated gaming. This approach is needed as most research in this field capitalized on variable-centered approach (e.g., predicting a dependent variable such as problematic gaming by a series of independent variables), thereby neglecting individual susceptibility influences (Rubin, 2009, Valkenburg and Peter, 2013). By including not only the characteristics of the players but also those of the games they prefer, such a person-centered perspective will align with and complement existing studies that relied on similar classification methods (Billieux et al., 2015b, Colder Carras and Kardefelt-Winther, 2018, Hussain et al., 2015, Kim and Kim, 2013, Larrieu et al., 2022, Larrieu et al., 2023). Adolescent-focused, multi-year longitudinal studies should be carried out to track adolescent gamers’ exposure to different game features and identify which ones appear to play a more crucial role in habit formation (where players engage out of routine rather than enjoyment or goal alignment) and the emergence of impaired control over gaming during this sensitive developmental period. Moreover, it will be essential to implement cross-cultural studies to compare how particular features (e.g., competitive rankings) influence the prevalence of dysregulated gaming in different cultural contexts (e.g., South Korea vs. the USA), and eventually understand potential culturally specific effects.

## 4 Conclusion

Although the relevance of design features of games in promoting diminished control over gaming has been increasingly acknowledged through the proposal of several taxonomical models, it remains important to advance empirical research in this area. Yet, and crucially, recommended research directions must shift from simplistic linear causal approaches (i.e., attributing dysregulated gaming to “addictive” game characteristics) to interactionist frameworks. This is because of the likelihood that players process features in unique ways based on their individual characteristics, motivations, psychological states, and contextual influences — each of which may mediate and moderate the effects of game design features, and interact with one another in a cumulative manner, to promote uncontrolled and problematic gaming. Further investigating dysregulated gaming patterns from a design feature perspective is pivotal to inform policy, prevention and intervention measures, as well as guide ethical design of video game products.

## CRediT authorship contribution statement

**Maèva Flayelle:** Writing – review & editing, Writing – original draft, Conceptualization. **Mélina Andronicos:** Writing – review & editing. **Daniel L. King:** Writing – review & editing. **Joël Billieux:** Writing – review & editing.

## Declaration of competing interest

The authors declare that they have no known competing financial interests or personal relationships that could have appeared to influence the work reported in this paper.

## Data Availability

No data was used for the research described in the article.
